# Toward More Inclusive Metrics and Open Science to Measure Research Assessment in Earth and Natural Sciences

**DOI:** 10.3389/frma.2022.850333

**Published:** 2022-03-28

**Authors:** Olivier Pourret, Dasapta Erwin Irawan, Najmeh Shaghaei, Elenora M. van Rijsingen, Lonni Besançon

**Affiliations:** ^1^UniLaSalle, AGHYLE, Beauvais, France; ^2^Applied Geology Research Group, Faculty of Earth Sciences and Technology, Institut Teknologi Bandung, Bandung, Indonesia; ^3^Central Administration, The University Library of Southern Denmark, Sønderborg, Denmark; ^4^Department of Earth Sciences, Utrecht University, Utrecht, Netherlands; ^5^Faculty of Information and Technology, Monash University, Clayton, MO, Australia

**Keywords:** Open Science, research assessment, inclusive metrics, award, h-index

“It will never be possible to harmoniously implement open science without a universal consensus on a new way of evaluating research and researchers.”

Bernard Rentier

## Introduction

**Figure 1:**
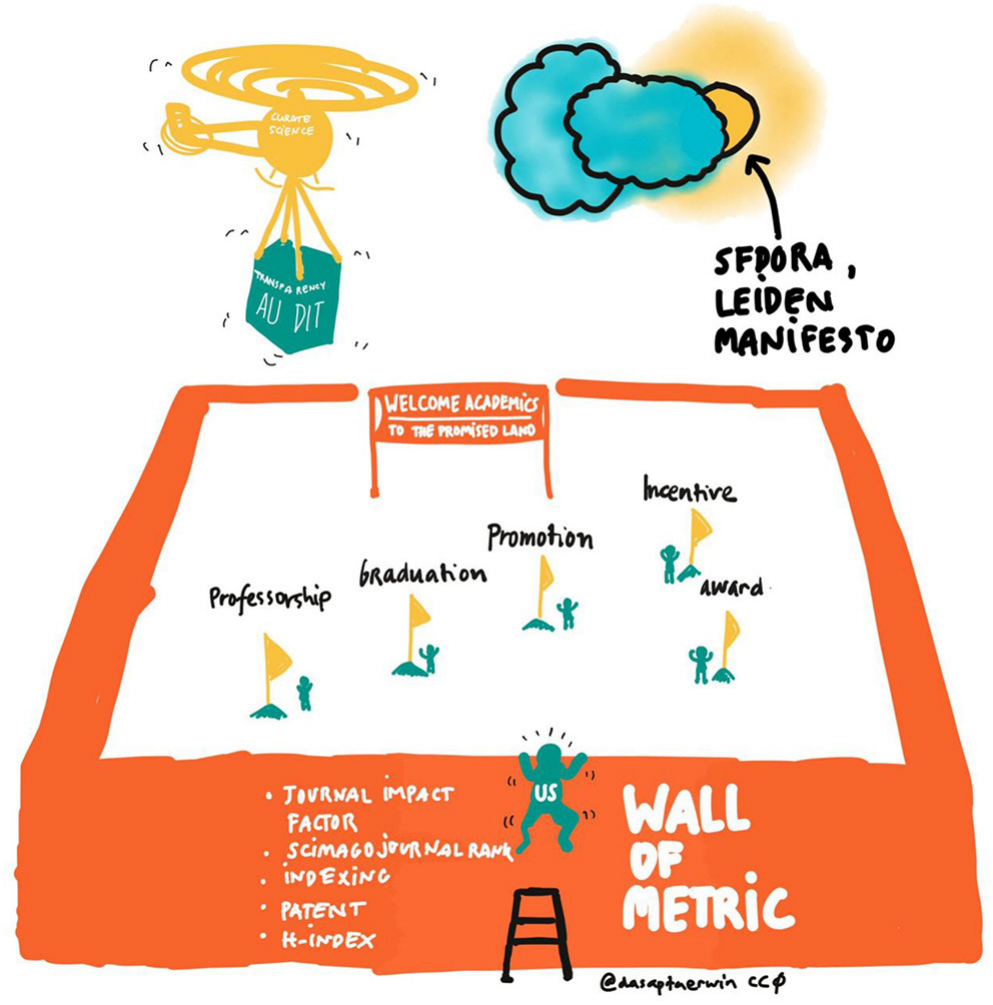
Sketchnote the “Wall of Metric” by Dasapta Erwin Irawan to showcase the small playground of researchers/scientists that is filled with self-centered indicators.

The conventional assessment of scientists relies on a set of metrics which are mostly based on the production of scientific articles and their citations. These metrics are primarily established at the journal level (e.g., the Journal Impact Factor), the article-level (e.g., times cited), and the author level (e.g., h-index; Figure 1). These metrics form the basis of criteria that have been widely used to measure institutional reputation, as well as that of authors and research groups. By relying mostly on citations (Langfeldt et al., 2021), however, they are inherently flawed in that they provide only a limited picture of scholarly production. Indeed, citations only count document use within scholarly works and thus provide a very limited view of the use and impact of an article. Those reveal only the superficial dimensions of a research's impact on society. Even within academia, citations are limited since the link they express does not hold any value (Tennant et al., 2019). As an example, one could be cited for the robustness of the presented work while the other could be cited for its main limitation (Aksnes et al., 2019). As such, two articles could be cited the same number of times for very different reasons, and relying on citations to evaluate scientific work therefore displays obvious limitations (Tahamtan et al., 2016). Beyond this issue, however, the conventional assessment of scientists is clearly beneficial to some scientists more than others and does not reflect or encourage the dissemination of knowledge back to the public that is ultimately paying scientists. This is visible in the Earth and natural sciences which has been organized to solve local community problems in dealing with the Earth system like groundwater hazards (Irawan et al., 2021; Dwivedi et al., 2022). Sadly, results of the conducted research rarely reach the public and dissemination often relies on volunteer efforts from scientists. The efforts bear close-to no weight in current scientific evaluation practices. The problem is even more present for scientists from Global South and/or non-English speaking countries. They carry heavier burdens of producing bilingual materials: (i) peer-reviewed articles in indexed reputable journals using high standard English- to satisfy current assessment methods and (ii) community outreach and engagement using local language to perform their responsibility to society (Irawan et al., 2021). However, the latter activity frequently lies on the bottom of their list given the already high workload necessary to publish peer-reviewed articles. This can be clearly observed by looking at the campaign launched by Asian and African universities showcasing their achievement in the World-Class University game. All publications are strongly encouraged to be written in English-language and assessments follow those typically drafted by, and beneficial for, western, educated, industrialized, rich, and democratic-nations (Gadd, 2021).

The limitations of traditional research assessment have been thoroughly demonstrated by scholars advocating for Open Science. They argue that our focus on citations and articles is both unfair and giving incentives for quantity over quality. Open Science is not a unified ideology but a diverse set of principles, practices, and goals (Besançon et al., 2021). Equity is often stated as a core aim of Open Science practice, but just because things are “open” will not necessarily ensure equity (Fecher and Friesike, 2014). Indeed, many factors like region, gender, discipline, and access to resources will continue to shape the possibilities of participation in an Open Science world (Davies et al., 2021). Public have been connecting Open Science only with Open Access (OA) journal publishing system with article processing charge (APC). Moreover, OA journals with APC (which mostly run by for profit publishers) have often been set as the standards of quality in OA publishing (especially in the SE Asia and also other regions). The question of “how” to fairly evaluate researchers using Open Science perspectives, however, remains a hot topic. Indeed, although Open Science aims to make all stages of research process (including evaluation) open and transparent, its limited role is an issue of practical implementation (a kind of challenge).

## Current Challenges

Beyond the limitations of the adopted metric-based evaluation of scientists, academia faces important and rising challenges that research assessment methods should consider.

(i) First, a significant barrier to greater engagement among scientists and researchers with stakeholders and community members is the persistent academic standard that productivity and impact be judged primarily by their productions of journal articles. Incentives for researchers to share their findings more broadly to policymakers, industry or to involve society in the process of research are generally quite limited and often not provided by funding agencies. There are funding programs whose evaluation criteria include dissemination, some do not. There is a strong reason for this difference: not all frontier science is easily accessible (or immediately relevant) to the public.

(ii) For science communication to be successful, professionals are required. Often it would be too much (if not impossible) to ask fundamental science researchers to engage in science communication—at least with a significant level of success. One could ask whether the role of science communicators deserves to become more established, but who should be in charge of such coordination and funding?

(iii) Funding agencies do some communication activities but not in a structured manner as to follow regular updates on specific topics (choices for featuring stories often fall on hot topics like global warming). This lack of a clear dissemination strategy results in many research findings and data remaining undelivered and untranslated and therefore inaccessible to policymakers, stakeholders, and the general public (Tennant and Wien, 2020).

(iv) Time effort and costs of publishing datasets, engaging the public, and communicating findings are proportionally greater for small projects, institutions, or research teams, putting even greater demands on these groups to achieve integrated, open, and networked science.

(v) A solution to this would be the use of alternative metrics (e.g., altmetrics; Pourret et al., 2020), or social metrics derived from research data dissemination and social outreach. The Metric Tide Report (https://responsiblemetrics.org/the-metric-tide/) make some proposals for alternatives to the impact factor.

(vi) Aside to those challenges mentioned above, another critically important, yet often disregarded factor, is the need for diversity in team composition. Representation among different genders, backgrounds, nationalities, and career stages can expand perspectives in a project (Nielsen et al., 2017). The system is now designed in such a way that it recognizes only one type of excellent scientist (the one with a high publication rate). Early Careers and promising scientists who do not recognize themselves in that profile might leave academia for that reason, leading to a loss of talent. A better definition of the various profiles/career paths of scientists will attract more diverse talents, as proposed in The Netherlands (https://recognitionrewards.nl/). Room is now being created for academics to include what they feel their strengths are and to focus on what matters most in their field. Academics will be given more opportunity to present their quality, content, academic integrity, creativity, and contributions to society (European Commission, Directorate General for Research Innovation, 2021), and then followed by the European Open Science Conference held in Paris early February 2022 and the Paris Call on research assessment (OSEC, 2022^1^; http://www.ouvrirlascience.fr/paris-call-on-research-assessment/).

The future decision to introduce new ways of recognizing and rewarding academics does not mean that the quality of research will be lower. In contrast, it is a positive choice for more team science: to promote multidisciplinarity, where one team member can be good at research, another at making an impact and yet another at teaching. The team will benefit collectively (see in geochemistry; Riches, 2022).

Local scientists and non-scientists can be great assets to projects, bringing valuable contextual information. However, even when researchers wish to engage communities and stakeholders, the approach taken can thwart community engagement efforts. Further, non-scientists are often dismissed by researchers, leading to disengagement by individuals who may bring great value to an effort. One factor that the science community must take into consideration is the collaboration between professional scientists and society. Such collaboration is a two-way process, which will empower non-scientists to play a role in research activity and produce improvements and make discoveries which will be of benefit to both parties (Ignat et al., 2018). Scientific journals and databases are less accessible to the general public and outreach is an effort to “translate,” simplify, and convey new scientific knowledge with the wider public community. Science communication in multiple non-English languages is also crucial for effective dissemination of scientific ideas (Márquez and Porras, 2020). However, journalists, who have taken the role of science dissemination, have a different educational background than scientists, resulting in difficult communication between them and potentially misinformation of the public. Therefore, we need a shift in the roles of both scientists and journalists.

Finally, the COVID-19 pandemic that limits physical interactions, has proven that conducting research in traditional closed mode (with articles published behind paywalls) also limits the collaboration and effectiveness of research development. But beyond this, it highlighted the crucial importance of fast knowledge dissemination (sometimes at the risk of misinterpreting) and community provided peer-review outside of traditional publishing and reviewing models; none of which are usually rewarded or considered by traditional scientific evaluation paradigm (Besançon et al., 2020). Peer-reviewers should not act as gate-keepers of science, instead they could take the role as the nurturers of science.

Overall, it thus seems that limitations of scientific evaluation are clearly apparent in all of the aforementioned challenges. None of these challenges are new and Open Science advocates have for long argued toward a radical change in scientific evaluation. It remains however disputed how scientific assessment should be undertaken in the future.

## Opportunities

Currently, there are several initiatives of the cross-stakeholder global movement to make sure that we are responsibly and appropriately assessing common values of higher education (scholarship) for the society, i.e., The Leiden Manifesto (http://www.leidenmanifesto.org/), the San Francisco Declaration on Research Assessment (DORA; https://sfdora.org/), or proposals to replace Journal Impact Factor. To date (March 2nd 2022), 21,303 individuals and organizations from 156 countries have signed DORA. Despite the strong campaign by Open Science advocates, the monitoring of the institutions which sign up for the manifestos would be difficult because Leiden Manifesto and DORA are based on self-assessment. Especially in some countries where the rate of institutional endorsement is low, there have been several high-level debates over which path to choose for national-level research assessments. As signatories of DORA ourselves, we advocate for removing journal impact factors in research assessment (Pourret, 2021). However, although many people would agree with the principles presented by DORA, publishing in top journals and projecting it as one's achievement is still in the daily conversation of academia (e.g., Nature Index). The efforts to track down researchers' publications in top journals and highly cited researchers have been used as an explicit race campaign at national or international levels, for example in Indonesia (Kemenristek/BRIN, 2020). In most assessment sheets in Indonesia, a community engagement, such as disaster preparedness coaching for junior high students living in the footslope of active Mount Agung in Bali (Saepuloh et al., 2018), will only be worth a tick mark. However, more researchers are also exposed to the principles of collaboration instead of competition (Pelupessy, 2017) that would bring a new fresh voice in the research ecosystem. The pandemic has presumably helped scientists collaborate more across the world, but we are also running into the digital divide (Irawan et al., 2020). In-person meetings have always been seen as the best method to communicate with each other, but virtual conferences have shown undeniable advantages (including accessibility), and we are convinced that the future of events will be hybrid (Pourret and Irawan, 2022).

More intentional engagement with local stakeholders, community members, and educators at the outset of a research effort has the potential to lead to more integrated, coordinated, and impactful Open Science (Goldman et al., 2021). It might also help in increasing scientific literacy (Garrison et al., 2021). During project inception and development, researchers should build in ways to involve stakeholders and society, ranging from defining scope and priorities of a research question based on community expertise to engaging the public in citizen science data collection. The American Geophysical Union's Thriving Earth Exchange provides a way for scientists to connect with communities seeking science support to resolve challenges that require the expertise of biogeochemistry (Dwivedi et al., 2022). Existing citizen science projects can provide ready-made infrastructure for engaging members of the public in data collection; thousands of such projects are listed at scistarter.org. Alternatively, researchers may create their own citizen science project leveraging existing infrastructure. In the context of reciprocity within Citizen Science, research libraries play a key role to support or engage in the projects, build skills for engaging, adopt toolkits or models, as well as promote positive attitude toward Citizen Science, thus creating an increased Public Understanding of Science (Overgaard and Kaarsted, 2018). To include social scientists, with skills and experience in engaging groups, can increase the chances that projects in Earth and natural sciences are designed with human dimensions and applications in mind and that products will be utilized by non-scientist audiences. With such engagement, we could expect a more fluid relationship between stakeholders of science. Publishing in more languages than English will help (Pourret and Irawan, 2022), point out the language barrier for non-English speaking countries to understand the content of English-written scholarship outputs. Creating secondary non-conventional outputs (e.g., a YouTube video or conversational podcast) to translate a paper written in English would, in a way, help more people to understand the content of the paper. One of several initiatives that further call to diversify language in scientific publications is Helsinki Initiative (https://www.helsinki-initiative.org/; Henry et al., 2021). It supports the dissemination of research output, while at the same time encourage in promoting local relevant research and the usage of local language.

Because much of the work carried out in the field of earth and natural sciences addresses issues intersecting with the environment, climate change, and biodiversity, these scientific disciplines are frequently covered in the news media. The benefits of such coverage are many, including a more informed public and demonstration of the return on public funds invested in the research. However, it is necessary to provide incentives for scientists to engage with the news media (Besançon et al., 2021) and to translate their work through less traditional mechanisms such as social media, blog posts, or videos. For that we need a dedicated structure that makes the process simple and easy.

As an example, the Danish Bibliometric Research Indicator (BFI) provides an overview of Danish research production. The BFI was introduced in 2009 to report the number of peer reviewed publications of each type that universities have produced (Deutz et al., 2021). The BFI model measures publications and publication channels degree of prestige, and indicates that quantitative analyses should never stand alone, but be supplemented by qualitative analyses. Indeed, even experienced researchers from the developed world publish in predatory journals mainly for the same reasons as do researchers from developing countries: lack of awareness, speed and ease of publication process, and a chance to get rejected work published. On the other hand, Open Access potential and larger readership outreach were also motives for publishing in open access journals with quick acceptance rates (Shaghaei et al., 2018). Moreover, another threats that take advantage of our assessment system, especially in the Global South, would be predatory journals (see discussion on definition in Grudniewicz et al., 2019) that are sometimes seen as a solution to wide dissemination of new research results, the COVID-19 pandemic has shown that preprints were actually more beneficial as they can also gather feedback (Fraser et al., 2021). However, they are not always considered as scientific output, but preprints are here to stay, and are valid scientific resources that deserve to be seen as scientific productions (Lanati et al., 2021), under specific conditions. Beyond the early and wide dissemination of scientific advances through preprints, the pandemic has also shown a need to recognize scientific communication and peer-review as the embedded parts of science implementation (e.g., outreach of COVID vaccination). Attitudes toward open peer review, open data, and use of preprints influence scientists' engagement with those practices. Further research is needed to determine optimal ways of increasing researchers' attitudes and their Open Science practices (Baždarić et al., 2021). While open peer-review could help ensure that credit is given where due, there are very few incentives for scientists to engage in thorough reviewing of their peers' work (Armani et al., 2021). Moreover, unseen work of early career researchers should be valued, indeed many of them ghost write peer-review in place of their principal investigator or mentor and are not dully rewarded for their work (McDowell et al., 2019).

Publishing a paper is one big effort, but creating engagement and tracking the impact of it is another huge effort that most researchers take for granted (Pourret et al., 2020). While increasing citations is one goal that people frequently mention when they are promoting their paper, creating engagement has more benefits than just adding some new citations to your portfolio (Ross-Hellauer et al., 2020).

## Concluding Remarks

Diversity, equity and inclusion are key components of Open Science. In achieving them, we can hope that we can reach a true Open Access of scientific resources, one that encompasses both (i) open access to the files (uploading them to a public repository) and (ii) open access to the contents (including language). Until we decide to move away from profit-driven journal-based criteria to evaluate researchers, it is likely that high author-levied publication costs will continue to maintain inequities to the disadvantage of researchers from non-English speaking and least developed countries. As quoted from Bernard Rentier, “the universal consensus should focus on the research itself, not where it was published.”

## Author Contributions

OP: conceptualization, writing—original draft, writing—review, and editing. DEI: visualization, writing—original draft, writing—review, and editing. NS, EMvR, and LB: writing—original draft, writing—review, and editing. All authors contributed to the article and approved the submitted version.

## Funding

This research was partly funded by the Science & Impacts grant awarded to the project Open Science in Earth Sciences by Ambassade de France en Indonésie.

## Conflict of Interest

The authors declare that the research was conducted in the absence of any commercial or financial relationships that could be construed as a potential conflict of interest.

## Publisher's Note

All claims expressed in this article are solely those of the authors and do not necessarily represent those of their affiliated organizations, or those of the publisher, the editors and the reviewers. Any product that may be evaluated in this article, or claim that may be made by its manufacturer, is not guaranteed or endorsed by the publisher.

## Footnotes

^1^ https://osec2022.eu/
